# DYN-1/dynamin regulates microtubule dynamics after axon injury.

**DOI:** 10.17912/micropub.biology.000549

**Published:** 2022-04-06

**Authors:** Tarika Vijayaraghavan, Samiksha Dhananjay, Brent Neumann

**Affiliations:** 1 Neuroscience Program, Monash Biomedicine Discovery Institute and Department of Anatomy and Developmental Biology, Monash University, Melbourne, Victoria 3800, Australia

## Abstract

Microtubules play essential roles in the regeneration of axons after injury, but precisely how their growth is regulated remains to be resolved. Here, we studied the influence of the *C. elegans* DYN-1/dynamin GTPase protein on microtubule growth after axon injury. Before injury, loss of DYN-1 had no effect on microtubule dynamics compared to wild-type animals. However, significant increases in microtubule dynamics were observed after axotomy in animals lacking DYN-1. Moreover, a greater proportion of these animals displayed microtubule growth in the retrograde direction compared to wild-type controls. These data establish a role for DYN-1 in regulating microtubule dynamics after injury in *C. elegans* .

**Figure 1. DYN-1 regulates microtubule dynamics in the PLM neuron.**
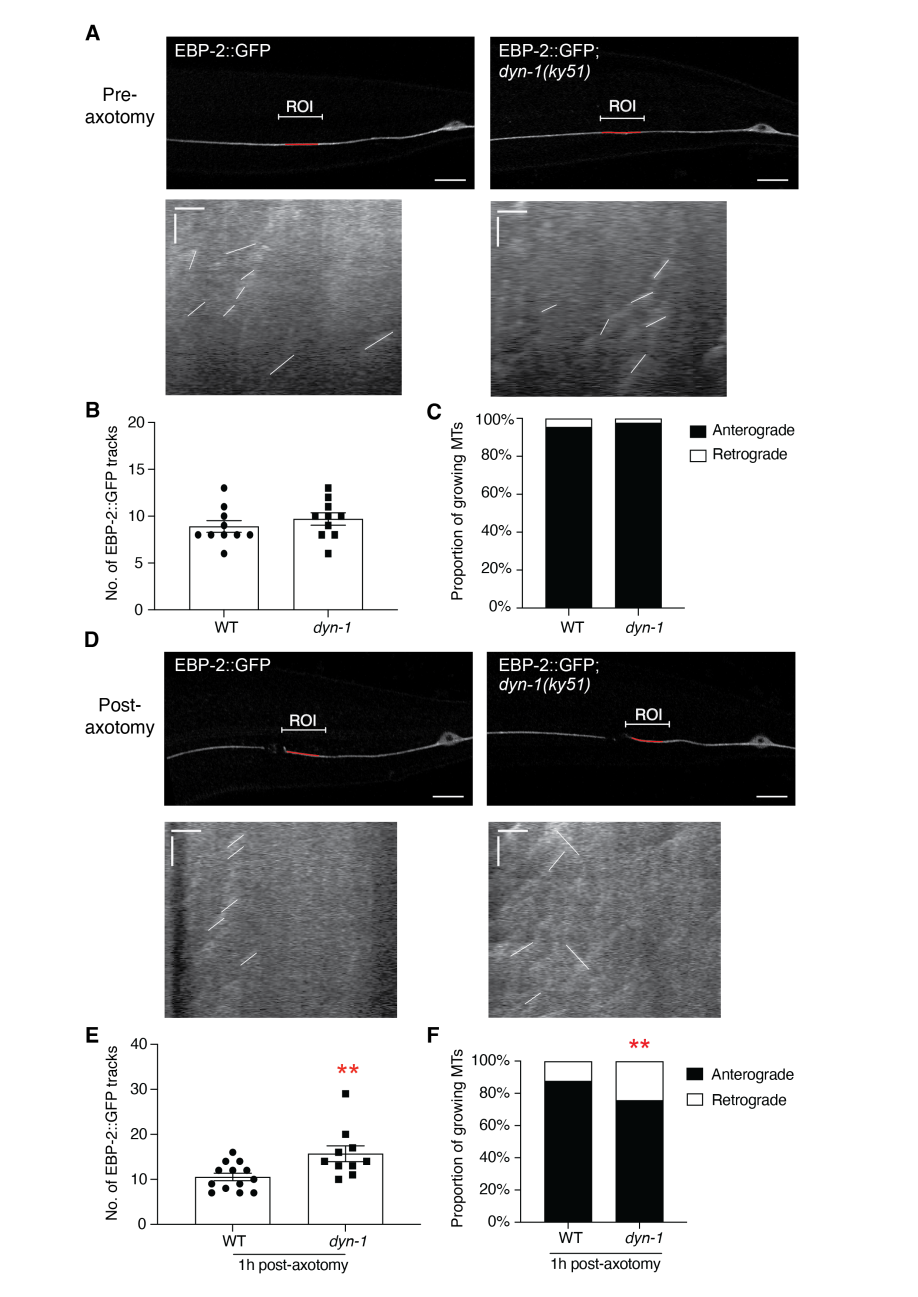
**(A)** Analyses of microtubule plus-end binding protein-2 (EBP-2) comets in WT (left panels) and *dyn-1(ky51)* (right panels) strains. Animals were incubated for at least 2 hours at 25°C before confocal imaging (top panels). Bars represent 10 µm. The corresponding kymographs (bottom panels) represent the movement of individual comets (white lines) over a distance of 14 - 15 µm (region of interest (ROI) shown in red). Horizontal and vertical bars represent approximately 2 µm and 20 seconds, respectively. **(B)** The number and **(C)** direction of EBP-2::GFP comets recorded in both WT and *dyn-1* animals pre-axotomy. **(D)** Analyses of individual EBP-2 comets measured 14-15 µm from the cut site (ROI shown in red) in WT and *dyn-1* animals 1-hour post-axotomy. The corresponding kymographs are shown below each image. Horizontal and vertical bars represent approximately 2 µm and 20 seconds, respectively. Animals were incubated at 25°C before and after axotomy. **(E)** The number and ( **F)** direction of EBP-2::GFP comets recorded in both WT and *dyn-1* animals post-axotomy. P-values calculated using unpaired student t-tests; **p<0.005, n > 9 worms or more than 100 comets per genotype.

## Description

The nematode *Caenorhabditis elegans* ( *C. elegans* ) is a tractable model organism in which to study the repair of severed neurons *in vivo* (Brenner 1974; Yanik et al. 2004; Chisholm et al. 2016; Neumann et al. 2019). Microtubules are major structural and functional components of the cytoskeleton that are also important for rebuilding axons after injury (Chen 2018; Blanquie and Bradke 2018). However, despite a wealth of information being gained into the importance of microtubules during axonal regeneration, we still lack a complete understanding of how their growth is regulated after injury.

The dynamin GTPase, which is most widely known for its role in endocytosis, has previously been shown to regulate microtubule dynamics in mammalian cells (Shpetner and Vallee 1989; Scaife and Margolis 1990; Shpetner and Vallee 1992; Herskovits et al. 1993). In this study, we examined whether the *C. elegans* DYN-1/dynamin protein influences microtubule growth after axon injury in the posterior lateral microtubule (PLM) neurons. To visualize microtubule dynamics, we imaged a GFP-tagged version of the microtubule end binding-2 (EBP-2) protein in the PLM neurons both before and after UV-laser induced axotomy (Chen et al. 2011; Ghosh-Roy et al. 2012; Neumann et al. 2014). Pre-axotomy imaging and analysis showed no visible difference between wild-type and *dyn-1(ky51)* mutant animals before axotomy (Figure 1 A-C). However, one hour after axotomy, microtubule dynamics were significantly increased in *dyn-1* mutants compared to wild-type animals (Figure 1 D-E). Additionally, post-axotomy analyses revealed a greater proportion of microtubule growth in the retrograde direction in *dyn-1* mutant animals compared to the wild-type (Figure 1 F), suggesting that DYN-1 is important for maintaining correct microtubule polarity during axonal regeneration. Together these data establish a role for DYN-1 in regulating microtubule dynamics post-injury in the *C. elegans* PLM neurons.

## Methods

Growth conditions: Hermaphrodites were used for all experiments. Animals were grown at 15°C and then transferred to 25°C for at least 2 hours before the pre-axotomy imaging. For analysis of the microtubule dynamics after axotomy, animals were incubated at 25°C both before and after axotomy. The BXN844 [ *dyn-1(ky51); juIs338(mec-4p::ebp-2::GFP + ttx-3p::RFP)* ] strain was used along with the CZ18975 [ *juIs338(mec-4p::ebp-2::GFP + ttx-3p::RFP)* ] strain (Ghosh-Roy et al. 2012; Chen et al. 2015). The *dyn-1(ky51)* allele is temperature sensitive, with 15°C representing the permissive temperature and 25°C the restrictive temperature.

Confocal microscopy: EBP-2::GFP dynamics were analyzed using a Zeiss LSM 980 confocal microscope (Zeiss Group, Oberkochen, Germany). Animals were immobilized in 0.05% tetramisole on freshly prepared 7-8% agarose in M9. Live imaging was performed at a frame rate of 630 ms with 1.59 frames captured per second for 200 frames. Analyses was performed using Fiji/ImageJ (version 1.0) wherein kymographs were generated to determine the number, direction and duration of EBP-2::GFP tracks. In intact PLM axons, EBP-2 tracks were traced for 14-15 µm starting approximately 35 - 40 µm from the cell body such that a similar region was examined in intact axons and in those that underwent axotomy. PLM axons were severed using a Micropoint UV laser system (Andor – Oxford Instruments, Belfast, UK) attached to Zeiss Axio Imager.A2 compound microscope (Zeiss Group, Oberkochen, Germany), approximately 50 μm from the cell body as described previously (Abay et al. 2017). In injured axons, EBP-2 tracks were manually traced 14-15 µm from the site of injury in the proximal fragment as there was no visible difference in the number of comets closer to the cell body between wild-type and *dyn-1* animals. Only tracks that were completely imaged and longer than 0.5 μm were analyzed.

Statistical analyses: Unpaired t-tests were used to compare the EBP-2::GFP tracks between *dyn-1(ky51)* animals and controls, where * *p* <0.05, ** *p* <0.005, *** *p* <0.001.
